# Deep Neural Networks for Depression Recognition Based on 2D and 3D Facial Expressions Under Emotional Stimulus Tasks

**DOI:** 10.3389/fnins.2021.609760

**Published:** 2021-04-23

**Authors:** Weitong Guo, Hongwu Yang, Zhenyu Liu, Yaping Xu, Bin Hu

**Affiliations:** ^1^School of Information Science Engineering, Lanzhou University, Lanzhou, China; ^2^School of Educational Technology, Northwest Normal University, Lanzhou, China; ^3^Gansu Provincial Key Laboratory of Wearable Computing, Lanzhou, China; ^4^National and Provincial Joint Engineering Laboratory of Learning Analysis Technology in Online Education, Lanzhou, China

**Keywords:** deep belief networks, facial expression, 3D deep information, affective rating system, depression recognition

## Abstract

The proportion of individuals with depression has rapidly increased along with the growth of the global population. Depression has been the currently most prevalent mental health disorder. An effective depression recognition system is especially crucial for the early detection of potential depression risk. A depression-related dataset is also critical while evaluating the system for depression or potential depression risk detection. Due to the sensitive nature of clinical data, availability and scale of such datasets are scarce. To our knowledge, there are few extensively practical depression datasets for the Chinese population. In this study, we first create a large-scale dataset by asking subjects to perform five mood-elicitation tasks. After each task, subjects' audio and video are collected, including 3D information (depth information) of facial expressions via a Kinect. The constructed dataset is from a real environment, i.e., several psychiatric hospitals, and has a specific scale. Then we propose a novel approach for potential depression risk recognition based on two kinds of different deep belief network (DBN) models. One model extracts 2D appearance features from facial images collected by an optical camera, while the other model extracts 3D dynamic features from 3D facial points collected by a Kinect. The final decision result comes from the combination of the two models. Finally, we evaluate all proposed deep models on our built dataset. The experimental results demonstrate that (1) our proposed method is able to identify patients with potential depression risk; (2) the recognition performance of combined 2D and 3D features model outperforms using either 2D or 3D features model only; (3) the performance of depression recognition is higher in the positive and negative emotional stimulus, and females' recognition rate is generally higher than that for males. Meanwhile, we compare the performance with other methods on the same dataset. The experimental results show that our integrated 2D and 3D features DBN is more reasonable and universal than other methods, and the experimental paradigm designed for depression is reasonable and practical.

## 1. Introduction

According to the World Health Organization (WHO), more than 350 million people of all ages suffer from depression disorder globally (Reddy, 2012). Depression (depressive disorder or clinical depression) is one of the most severe but prevalent mental disorders globally. Depression can induce severe impairments that interfere with or limit one's ability to conduct major life activities for at least 2 weeks. During at least 2 weeks, there is either a depressed mood or a loss of interest or pleasure, as well as at least four other symptoms that reflect a change in functioning, such as problems with sleep, eating, energy, concentration, self-image, or recurrent thoughts of death or suicide. Depression can occur at any age, and cases in children and adolescents have been reported^1^. Because of its harmfulness and recent prevalence, depression has drawn increasing attention from many related communities.

Although it is severe, depression is curable through medication, psychotherapy, and other clinical methods (National Collaborating Centre for Mental Health, 2010). The earlier that treatment can begin, the more effective it is. Thus, the early detection of depression is critical to controlling it at an initial stage and reducing the social and economic burden related to this disease. Traditional diagnosis approaches for depression are mostly based on patients self-reporting in clinic interviews, behaviors reported by relatives or friends, and questionnaires, such as the Patient Health Questionnaire (PHQ-9) (Kroenke and Spitzer, 2002) and the Beck Depression Inventory (BDI-II) (McPherson and Martin, 2010). However, all of them utilize subjective ratings, and their results tend to be inconsistent at different times or in different environments. During the diagnosis, several clinical experts must be involved to obtain a relatively objective assessment. As the number of depressed patients increases, early-stage diagnosis and re-assessments for tracking treatment effects are often limited and time consuming. Therefore, machine learning-based automatic potential depression risk detection or depression recognition is expected to facilitate objective and fast diagnosis to ensure excellent clinical care quality and fundamentally reduce potential harm in real life.

Under the influence of depression, behavior disorder-based signals for depression recognition are increasingly extensive, such as voices (Ooi et al., 2013; Yang et al., 2013; Nicholas et al., 2015; Jiang et al., 2017), facial expressions (Schwartz et al., 1976; Babette et al., 2005), gestures (Alghowinem et al., 2018), gaits (Michalak et al., 2009; Demakakos et al., 2015), and eye movements (Winograd-Gurvich et al., 2006; Alghowinem et al., 2013; Carvalho et al., 2015). This work focuses on using facial expressions to recognize patients with potential depression risk. The research on depression based on facial expression essentially utilize video or images (Gupta et al., 2014; Alghowinem, 2015; Pampouchidou et al., 2015, 2016a; Bhatia et al., 2017). To be more precise, the interests are localized on images, facial landmark points (Stratou et al., 2014; Morency et al., 2015; Nasir et al., 2016; Pampouchidou et al., 2016b), and/or facial action units (AUs) (Cohn et al., 2009; McIntyre et al., 2009; Williamson et al., 2014). However, the methods that adopt image analysis (the essence of the video-based method are still images analysis where videos are converted into images) are affected by environmental factors and instrument parameters, such as illumination, angle, skin color, and resolution power. If these factors are not addressed appropriately, the recognition performance will be affected. Several researchers (Gong et al., 2009; Zhao et al., 2010) proposed using in-depth information captured from 3D sensors, which is relatively illumination, angle, and skin color invariant. However, 3D points of information can lose the texture features of facial expression. Therefore, the fusion of 2D with 3D data can make up for each other to address these issues.

Depression recognition typically comprises two steps: feature extraction and recognition (depression or not/ depression severity). The quality of feature extraction directly affects the result of recognition. Conventional feature extraction methods for depression facial expression utilize geometric features, appearance features, and dynamics. These methods extract the displacement of facial edges, corners, coordinates (McIntyre et al., 2010; Bhatia et al., 2017), mean squared distance of all mouth landmarks to the mouth centroid (Gupta et al., 2014), and displacement from the mid-horizontal axis to depict the changes and intensity of basic expressions (Bhatia, 2016). The local binary pattern (LBP), LBP-TOP (Joshi et al., 2012), local Gabor binary pattern (LGBP-TOP) (Sidorov and Minker, 2014), local curvelet binary pattern (LCBP-TOP) (Pampouchidou et al., 2015), and LPQ from three orthogonal planes (LPQ-TOP) (Wen et al., 2015) extracted describe the texture changes in the facial region. Histogram of optical flow (Gupta et al., 2014), motion history histogram (MHH) (Meng et al., 2013), and space-time interest points (STIPs) (He et al., 2015) are extracted to describe the facial motion. These results indicated that depressed people display a lower performance when responding to positive and negative emotional content. Nevertheless, all those mentioned approaches are hand-crafted feature descriptors designed based on tremendous professional knowledge, and image processing is also complicated for hand-crafted features. However, our cognition of depression remains insufficient. Such features probably yield segmented representations of facial expressions and are insufficiently discriminative. Simultaneously, the dynamics are extracted from a video, which involves the effect of the environmental factors mentioned above. On the other hand, the time window is used to extract motion features (Pampouchidou et al., 2016a; He et al., 2018). The reported window lengths are 60 frames, 20 frames, 5 frames, or even 300 frames. However, the optimal window length cannot be determined because there are significant variations over time in the facial expression according to the particular person and experimental device.

In recent years, deep learning techniques have prevailed in audio- and video-based applications, especially in visual information processing (Girshick et al., 2014). The purpose of this study is to identify the patients at risk of depression. The selected subjects are outpatients, and the evaluated depression degree is moderate. Many samples with depression risk and the normal control group had no noticeable expression changes in some stimulation tasks. Therefore, we chose the generative model deep belief network (DBN). The DBN-based deep learning method can hierarchically learn good representation from original data; thus, the learned facial features should be more discriminative than hand-crafted features for depression recognition. Long short-term memory (LSTM) is an effective and scalable model for learning problems related to sequential data and can capture long-term temporal dependencies. Facial expression is a dynamic process of continuous change, and it is a time-series signal on a timeline. Then facial expression motion is captured by LSTM used on the entire timeline.

The availability of clinical data is critical for the evaluation of methods for depression recognition. Because of the sensitivity of clinical data and privacy reasons, datasets for depression research are neither extensive nor free. It is why most research groups resort to generating their datasets. The current datasets are as follows: Pittsburgh, BlackDog, DAIC-WOZ, AVEC, ORI, ORYGEN, CHI-MEI, and EMORY, but only three of which are available. AVEC is the only fully public dataset available for free download, DAIC-WOZ is partly available, while Pittsburgh is also available, but not accessible now. The rest depression-related datasets are proprietary, and the corresponding research results are few. The securable datasets above provide the third-parties visual and audio features. Only AVEC discloses complete video recordings. However, these datasets are collected from non-Chinese subjects, which differ from Chinese subjects in terms of emotional expressiveness due to different cultural backgrounds. Thus, we used a structured experimental paradigm to construct a depression database specifically for Chinese subjects in conjunction with relevant psychiatric hospitals. To the best of our knowledge, the database we have established is the only database with complete data, a reasonable structure, and the largest number of subjects in China. Our dataset includes complete video recordings from typical webcam and microphone, and 3D 1,347 facial points scan from deep camera Kinect (Leyvand et al., 2011). Not only does a Kinect detect the human face, but it also provides real-time access to over 1,000 facial points in the 3D space irrespective of the color of the skin or the surrounding environment, illumination, or distance from the camera.

This paper builds on our previous work (Guo et al., 2019) by adding 2D static image information and 3D facial point motion information to identify depression, and it is a further improvement and summary of the original work. We build two different deep networks, respectively, one of which extracts static appearance feature using 2D images based on DBN, and the other learns the facial motion via 3D facial landmark points and facial AUs using DBN-LSTM. The two kinds of deep networks are then integrated by joint fine-tuning, which can further improve the overall performance. Therefore, our main contributions in this paper can be summarized as follows:

1. We designed a reasonable and effective experimental paradigm, collected diversified data and three kinds of samples (normal population, outpatients, and inpatients) combined with specialized hospitals, and constructed a large-scale dataset for depression analysis.
2. The two deep networks proposed can extract appearance features from 2D images and motion features from 3D facial landmark points. The integrated networks can achieve the fusion of static and dynamic features, which can improve recognition performance.
3. We have proved qualitatively and quantitatively that depressive prone groups show significant differences from healthy groups under positive and negative stimuli.

The following section briefly describes the related works on depression recognition based on facial expression. In section 3, we introduce the proposed depression recognition network structure. Dataset creation, experimental setting, results, and analysis are reported in section 4. Finally, some discussions and future works are provided in section 5.

## 2. Related Work

### 2.1. Depression Recognition Based on Machine Learning

Machine learning tools for depression detection have access to the same streams of information that a clinician utilizes for diagnosis. For example, the variation of facial expression, gesture, voice, and language should occur in communication modality. Reduced emotional expression variability is commonly found in depression and connected with deficits in expression positive and negative emotion (Rottenberg et al., 2005). In the following, we briefly summarize some excellent research results on identifying depression from visual cues.

Wang et al. (2008) extracted geometric features from 28 regions formed by 58 2D facial landmarks to characterize facial expression changes. Probabilistic classifiers were employed to propagate the probabilities frame by frame and create a probabilistic facial expression profile. The results indicate that depressed patients exhibit different trends of facial expressions than healthy controls. Meng et al. (2013) employed motion history histogram (MHH) to capture motion information of facial expression. Local binary patterns (LBP) and edge orientation histogram (EOH) features were then extracted, and partial least square (PLS) was finally applied for prediction. These features were extracted from images. Nasir et al. (2016) employed perceptually motivated distance and area features obtained from facial landmarks to detect depression. The window-based representation of features was used to capture large-scale temporal contexts results. Anis et al. (2018) developed an interpretable method of measuring depression severity. Barycentric coordinates of facial landmarks and rotation matrix of 3D head motion were used to extract kinematic features, and a multi-class SVM was used to classify the depression severity.

The methods mentioned above are based on traditional machine learning methods to extract hand-crafted facial expression feature descriptors for depression analysis. Some studies have also utilized deep learning to extract high-level semantic features of facial expressions from raw video recordings for automatic depression detection. Jan et al. (2018) utilized convolutional neural networks (CNN) to extract many different visual primitive features from the facial expression frames, while feature dynamic history histogram (FDHH) was employed to capture the temporal movement on the features. Zhou et al. (2020) presented a DCNN regression model with a GAP layer for depression severity recognition from facial images. Different face regions were modeled, and then these models were combined to improve the overall recognition performance. The results indicated that the salient regions for patients with different depression levels were usually around the eyes and forehead. Melo et al. (2019) used two 3D CNNs to model the spatiotemporal dependencies in global and local facial regions captured in a video, and then combine the global and local 3D CNNs to improve the performance. The CNN-based method mentioned above requires a large amount of data to train the model. Once the amount of data is small, it is easy to fall into overfitting. By comparing the data volume of existing studies with our method, we found that the state-of-the-art research used about 4,350 min of video-based publicly available datasets, while the amount of video data we used was only about 2,080 min. Existing studies have shown that the generation model has a better classification effect than the discriminant model in low samples (Ng and Jordan, 2002). So, we finally choose to use the DBN model.

### 2.2. DBN

The DBN (Hinton et al., 2006) is a generative model that uses multiple layers of feature-detecting neurons. It can learn hierarchical representation from raw input data and can be effectively built by stacking a restricted Boltzmann machine (RBM) (Fischer and Igel, 2012) layer-by-layer and greedily training it. In our study, the Gaussian–Bernoulli RBM is adopted to use real-valued visible units to train the first layer of the DBN; binary hidden units are used for training the higher layers. For a Gaussian–Bernoulli RBM, the energy function of a joint configuration is given as Equation (1).

(1) $$\begin{array}{r} \text{E}\left(\text{V},\text{H}\right)=\frac{1}{2\sigma^{2}}\overset{m}{\underset{i=1}{\sum }}\frac{{\left(v_{i}-a_{i}\right)}^{2}}{2}-\frac{1}{\sigma^{2}}\left(\overset{m}{\underset{i=1}{\sum }}\overset{n}{\underset{j=1}{\sum }}w_{ij}v_{i}h_{j}+\overset{m}{\underset{j=1}{\sum }}b_{j}h_{j}\right) \end{array}$$

where *a* ∈ *R*^*D*^ and *b* ∈ *R* are the biases for visible and hidden units, respectively. *w*_*ij*_ ∈ *R* is the weight between the visible unit *i* and the hidden unit *j*, while *m* and *n* are the numbers of visible and hidden units, respectively. σ is a hyper-parameter. As there are no connections between units in the same layer, the conditional probability distributions are given by Equations (2) and (3).

(2) $$\begin{array}{r} \text{P}\left(h_{j}=1|v\right)=\mathrm{sigmoid}\left(\frac{1}{\sigma^{2}}\left(\overset{m}{\underset{i=1}{\sum }}w_{ij}h_{j}+b_{j}\right)\right) \end{array}$$

(3) $$\begin{array}{r} \text{P}\left(v_{i}|h\right)=N\left(a_{i}+\overset{n}{\underset{j=1}{\sum }}w_{ij}h_{j},\sigma^{2}\right) \end{array}$$

where N(μ,v) is a Gaussian function with mean μ and variance *v*. (*w, a, b*) are the parameters of the RBM and are learned using contrastive divergence. The generated features are the posteriors of the hidden units in the case of given visible units. Finally, the top output values are classified using sigmoid activation and the stochastic gradient descent method is used to train the deep networks.

### 2.3. LSTM

**Figure 1:**
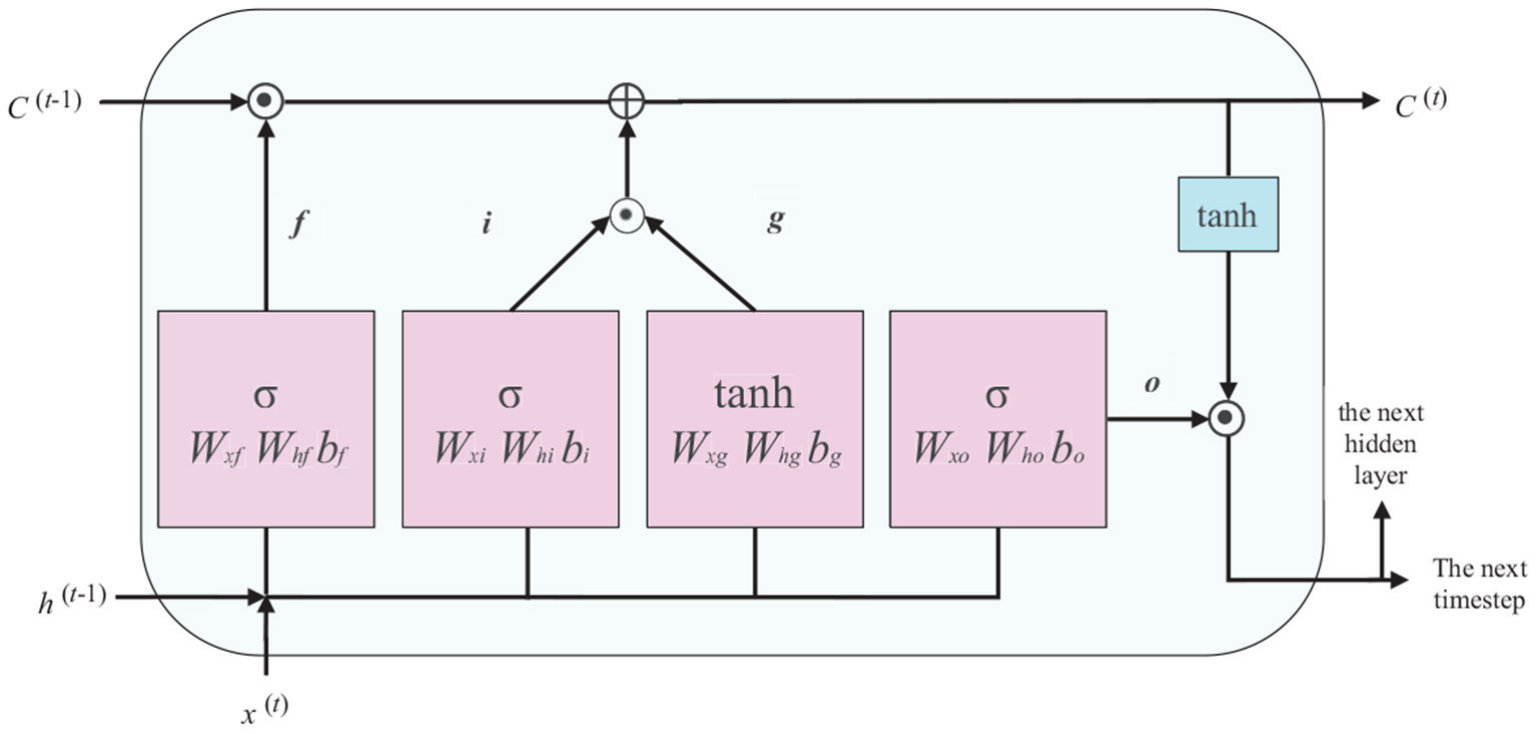
Deployment structure of long short-term memory (LSTM) unit.

The LSTM block has a memory cell that stores information with long-term dependencies. We use an LSTM with a conventional structure, as shown in Figure 1.

In Figure 1, *x*^(*t*)^ is the input data in time step *t* (the current frame), *h*^(*t*−1)^ is the hidden unit in time step *t*−1 (the previous frame), and *C*^(*t*−1)^ represents the cell status of the previous time step, which is modified to obtain the cell status of the current timestep *C*^(*t*)^. The flow of information in the LSTM is controlled by the computing unit described in Figure 1, namely the forget, input, and output gates. The specific process is described by the following equations:

*forget gate* *f*_*t*_*:* control the retention and removal of features.

(4) $$\begin{array}{r} f_{t}=\mathrm{sigmoid}\left(W_{xf}x^{\left(t\right)}+W_{hf}h^{\left(t-1\right)}+b_{f}\right) \end{array}$$

*input gate* *i*_*t*_*:* update cell status with input node *g*_*t*_.

(5) $$\begin{array}{r} \begin{array}{c} i_{t}=\mathrm{sigmoid}\left(W_{xi}x^{\left(t\right)}+W_{hi}h^{\left(t-1\right)}+b_{i}\right)\\ g_{t}=\tanh\left(W_{xg}x^{\left(t\right)}+W_{hg}h^{\left(t-1\right)}+b_{g}\right)\\ C_{t}=\left(C^{\left(t+1\right)}⊙f_{i}\right)⊕\left(i_{t}⊙g_{t}\right) \end{array} \end{array}$$

*output gate* *O*_*t*_*:* update the value of a hidden unit.

(6) $$\begin{array}{r} \begin{array}{c} O_{t}=\mathrm{sigmoid}\left(W_{xo}x^{\left(t\right)}+W_{ho}h^{\left(t-1\right)}+b_{o}\right)\\ h_{t}=O_{t}⊙\tanh\left(C^{\left(t\right)}\right) \end{array} \end{array}$$

where the weight matrix subscripts have the obvious meaning. For example, *W*_*hf*_ is the hidden-forget gate matrix, and *W*_*xi*_ is the input-input gate matrix. The bias terms *b*, the subscripts of *f*,*i*,*g*, and *o*, denote the corresponding door's bias.

### 2.4. Problem Setup

We find that the various kinds of effective methods proposed are based on 2D images (video is split into images) and 2D landmark point data (extracted from 2D images) by a survey of the current research on depression based on visual cues. The main limitations of 2D image-based analysis are problems associated with large variations in pose, illumination, angle, skin color, and resolution power. Nevertheless, depth information captured from 3D sensors is relatively posed and illumination invariant. Inspired by the idea of Aly et al. (2016), the fusion of 2D with 3D data can address these issues and cover the shortage of each other that 3D landmark points miss texture feature. Each expression can be decomposed into a set of semantic AUs, which exhibit in different facial areas and at different times with different intensities. Therefore, the dataset we build contains both 2D video and 3D landmark points and AUs information. In the paper, we propose a novel approach for depressive prone patients recognition based on two kinds of different DBN models combination, one of which extracts 2D appearance features from facial images collected by optical cameras, the other learns the facial motion from 3D facial points and facial AUs collected by a Kinect. The final decision result comes from the combination of the two networks. Finally, we evaluate all proposed deep models in our built dataset and analyze three aspects: gender, stimulus task, and affective valence.

## 3. Proposed Approach

### 3.1. The Framework of Deep Neural Networks-based Depression Recognition

**Figure 2:**
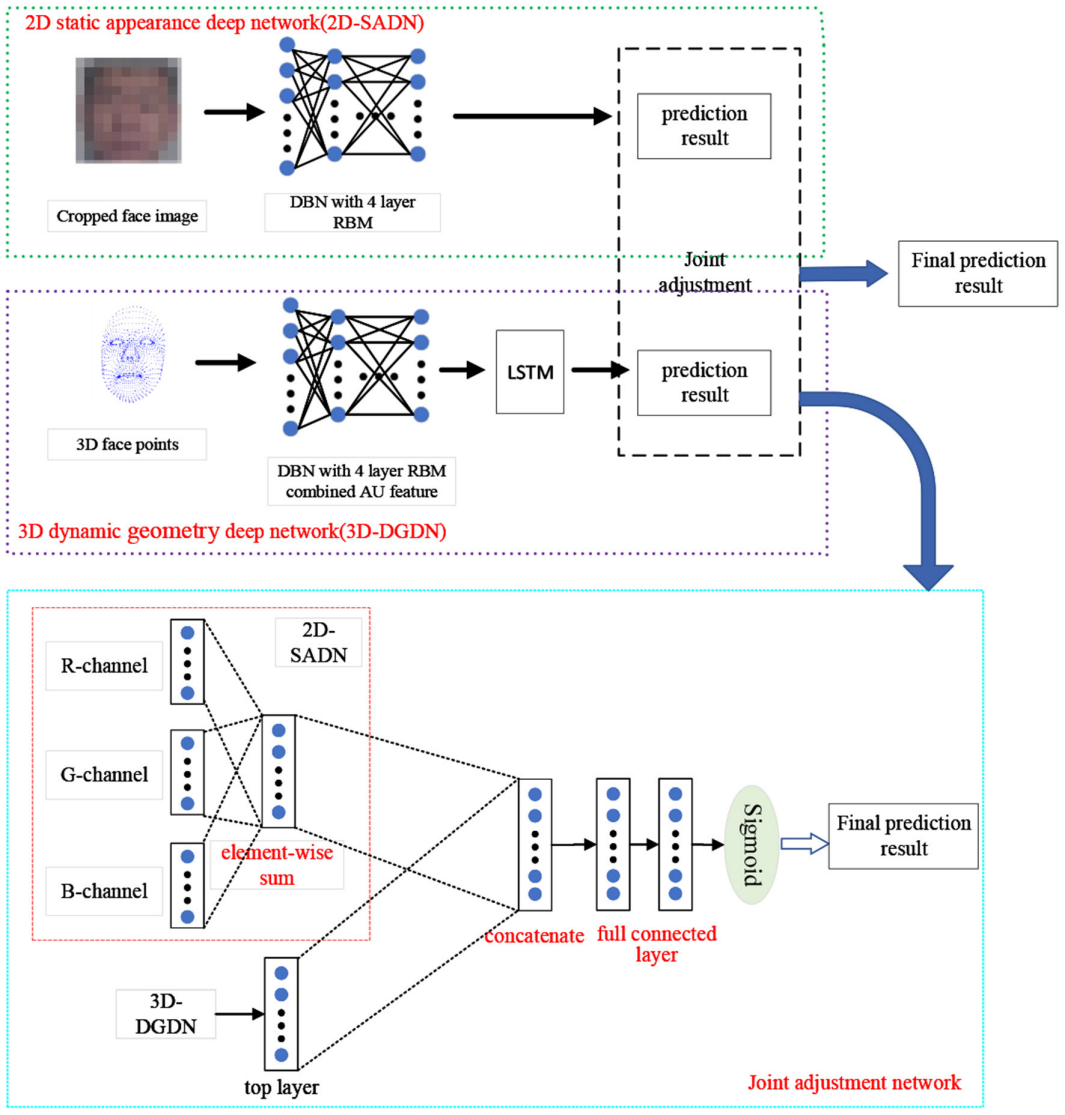
The framework of proposed approach.

We utilize the DBN and LSTM to potential depression risk recognition. We build two different deep networks: 2D static appearance deep network (2D-SADN), which is used to extract the static appearance features from images based on DBN. In other words, the network only focused on the analysis of appearance from static facial pictures in which a single image was used as input to the network and the network structure did not encode temporal information. 3D dynamic geometry deep network (3D-DGDN) based on combined of DBNs and LSTM, which capture the dynamic geometry features of 3D facial landmark points and AUs from Kinect. Expressions are inherently dynamic events consisting of onset, apex, and offset phases (Liu et al., 2014). Therefore, in the second network, we took the facial contour map composed of facial landmark points as input and used the position offset of the three-dimensional coordinate value on the time axis to obtain motion information. Finally, the two networks are integrated to improve the recognition performance. The overview of the proposed approach is shown in Figure 2.

#### 3.1.1. The Structure of DBN Model

**Figure 3:**
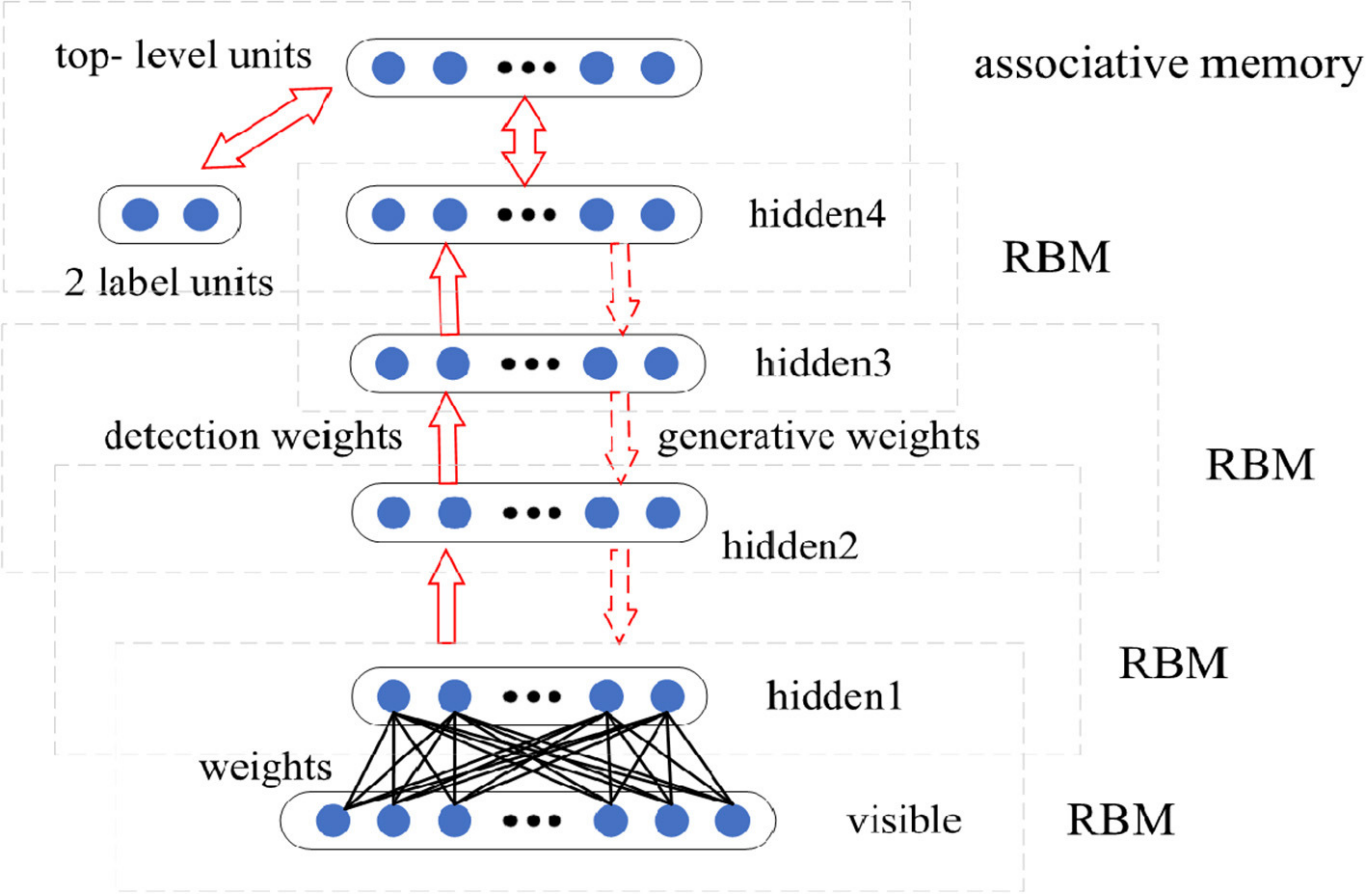
The structure of designed deep belief network (DBN) model.

The designed basic DBN network is composed of four RBMs, as shown in Figure 3. First, Gibbs sampling and contrastive divergence are adopted to train RBM to maximize *E*_*V*~_*p*__data__log*p*(*v*). The RBM parameter defines the parameters of the first layer of the DBN. Then, the second RBM is trained to approximately maximize $E_{V\sim p_{\text{data}}}E_{h^{\left(1\right)}\sim p^{\left(1\right)}\left(h^{\left(1\right)}|v\right)}\log p^{\left(2\right)}\left(h^{\left(1\right)}\right)$, where *p*^(1)^ is the probability distribution represented by the first RBM, and *p*^(2)^ is the probability distribution represented by the second RBM. That is, the second RBM is trained to simulate the distribution defined by the hidden unit sampling of the first RBM, which is driven by the input data. This process is repeated four times to add four hidden layers to the DBN, and each new RBM models the samples of the previous RBM. Each RBM defines another layer of DBN. Top-down fine-tuning is used to generate weights to guide the determination of the DBN model. At the top two levels, the weights are linked together so that the output of the lower level will provide a correlation to the top level, which will then link it to its associative memory. DBN can adjust the discriminant performance by using the labeled data and BP algorithm.

#### 3.1.2. Learning the Static Appearance Deep Network

**Figure 4:**
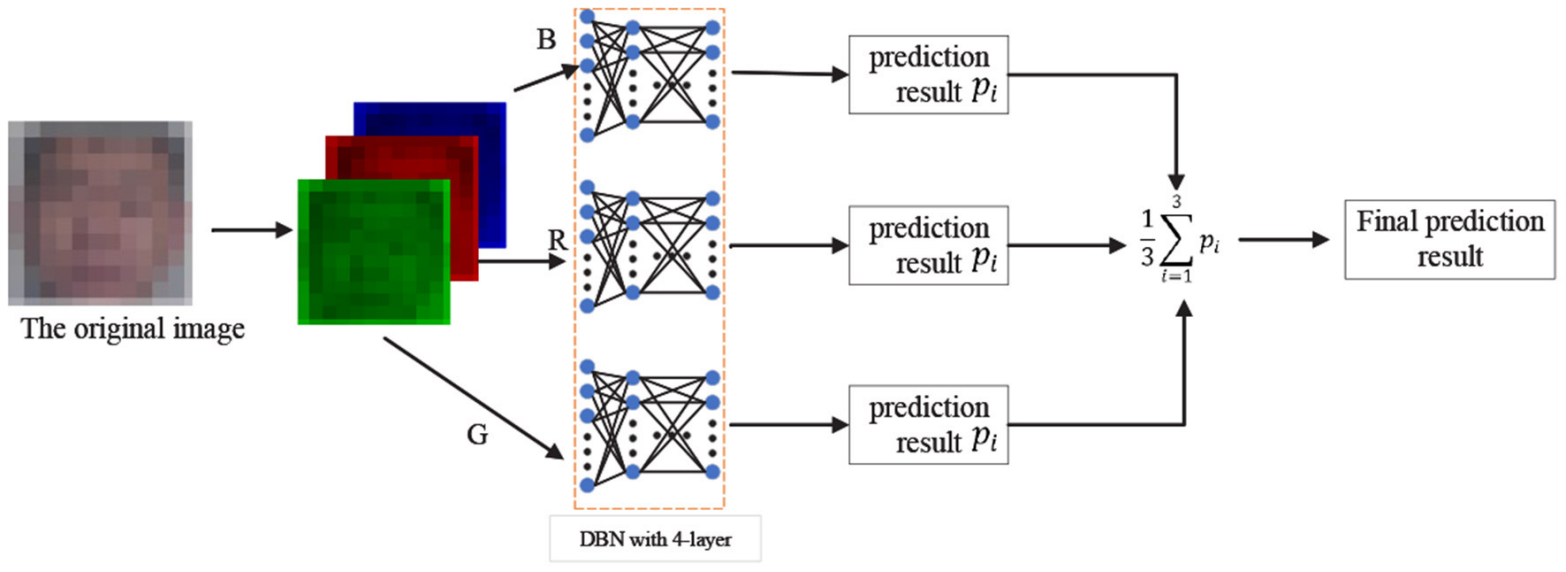
2D static appearance deep network.

In the 2D-SADN, we train a DBN as shown above with four layers by oneself with three channels, and then average the predicted values of each channel. The result is the final predicted value, as shown in Figure 4.

#### 3.1.3. Learning the Dynamic Geometry Deep Network

**Figure 5:**
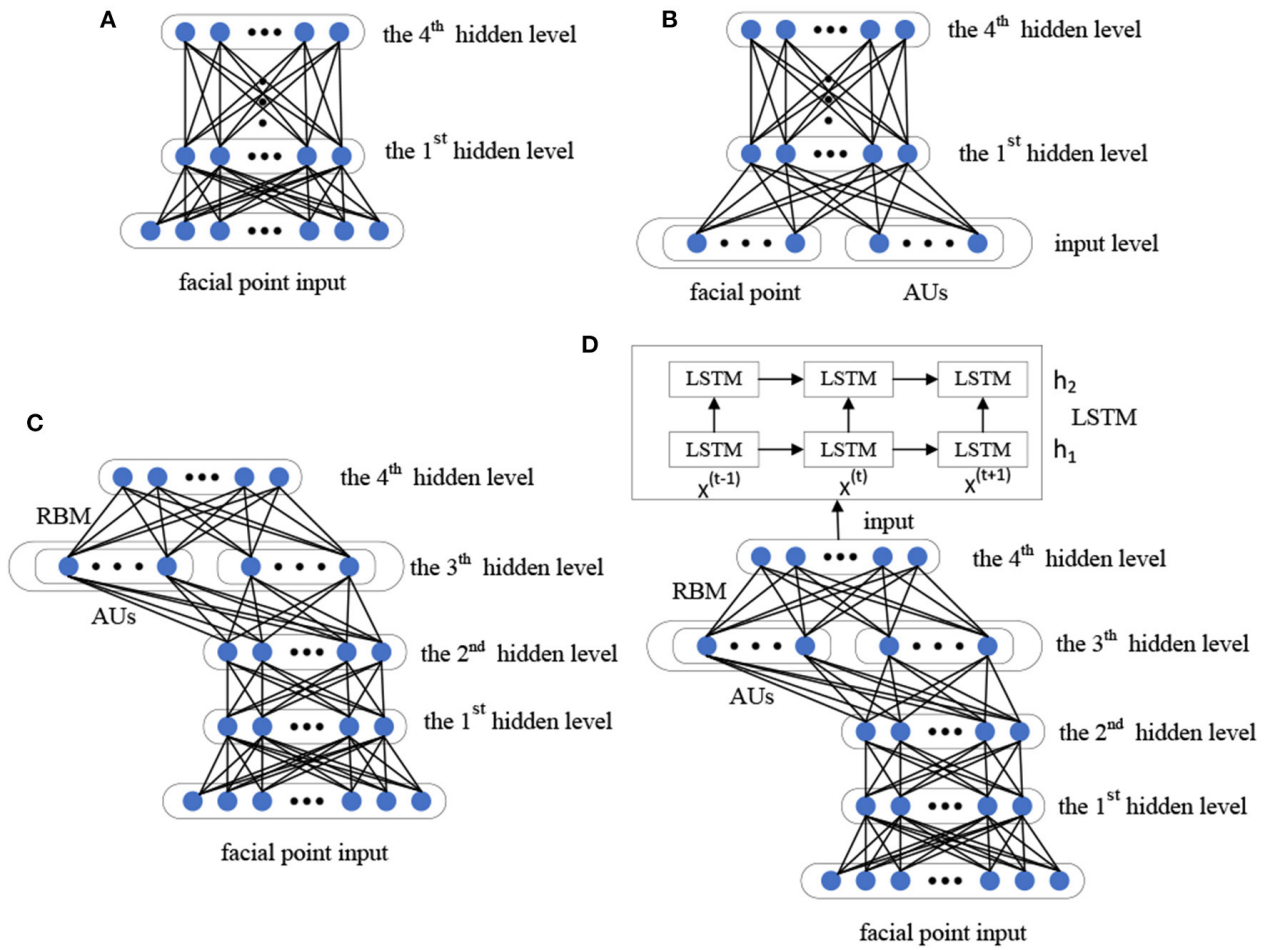
Framework of four different deep belief network (DBN) models: **(A)** 4DBN, **(B)** 4DBN-AU, **(C)** AU-4DBN, **(D)** AU-4DBN-LSTM.

In the 3D dynamic geometry deep network (3D-DGDN), we build four different DBN models based on our designed basic DBN network, as shown in Figure 5. Figure 5A is a four-hidden layer DBN with facial points, named 4DBN; Figures 5B,C shows four hidden layer DBNs with facial points and AU, named AU-4DBN and 4DBN-AU. Figure 5D shows a four hidden layer DBNs with facial points and AU followed by a LSTM, named AU-4DBN-LSTM. In the meantime, we build a five-hidden-layer DBN using facial points as the input and find that the accuracy rate of the four-hidden layer-DBN is almost the highest in all of the stimulus tasks, therefore a four-hidden-layer network is used as the basic DBN structure and AU-4DBN-LSTM stands for 3D-DGDN. The details are as follows.

- *4DBN* is a four-hidden layer DBN only using facial points as the input, as shown Figure 5A.
- *4DBN-AU* is a four-hidden layer DBN based on 4DBN that uses AU and facial points as the input, as shown Figure 5B.
- *AU-4DBN* is a four-hidden-layer DBN with AU added at the penultimate layer, which is used as the input of stacking an extra RBM on the top, as shown in Figure 5C.
- *AU-4DBN-LSTM* is based on the AU-4DBN model to add the LSTM. That is, the output of the RBM on the top of AU-4DBN is used as the input to the first layer of the LSTM (Greff et al., 2016), which has two layers, as shown in Figure 5D.

#### 3.1.4. Joint Fine-turning Method

The strategy of adding the corresponding position elements was used to connect the activation values of the top hidden layers of three channels to obtain the feature vector in the 2D-SADN. We then concatenated the feature vector and the activation values of the top hidden layers of 3D-DGDN. Finally, the concatenated values are used for inputs to a fully connected network with a sigmoid activation, as shown in Figure 2. Consequently, we integrate the network using a linear weighted sum of the loss function based on Jung et al. (2015), defined as follows:

(7) $$\begin{array}{r} L_{fusion}=\lambda_{1}L_{2D-SADN}+\lambda_{2}L_{3D-DGDN}+\lambda_{3}L_{2D-3D} \end{array}$$

where *L* is cross entropy loss function, and *L*_2*D*−*SADN*_, *L*_3*D*−*DGDN*_, and *L*_2*D*−3*D*_ are computed by 2D-SADN, 3D-DGDN, and the both, respectively. λ_1_, λ_2_, and λ_3_ are turning parameters. In order to fully utilize the capabilities of the two models, we set the value of λ_1_, λ_2_, and λ_3_ to 1, 1, and 0.1, respectively. Cross entropy loss function is defined as follows:

(8) $$\begin{array}{rc} L_{2D-SADN} & =-\overset{n}{\underset{i=1}{\sum }}y^{\left(i\right)}\log{ŷ}_{2D-SADN}^{\left(i\right)}\\ \;+\left(1-y^{\left(i\right)}\right)\log\left(1-{ŷ}_{2D-SADN}^{\left(i\right)}\right) \end{array}$$

(9) $$\begin{array}{rc} L_{3D-DGDN} & =-\overset{n}{\underset{i=1}{\sum }}y^{\left(i\right)}\log{\hat{y}}_{3D-DGDN}^{\left(i\right)}\\ \;+\left(1-y^{\left(i\right)}\right)\log\left(1-{ŷ}_{3D-DGDN}^{\left(i\right)}\right) \end{array}$$

(10) $$\begin{array}{rc} L_{2D-3D} & =-\overset{n}{\underset{i=1}{\sum }}y^{\left(i\right)}\log{ŷ}_{2D-3D}^{\left(i\right)}+\left(1-y^{\left(i\right)}\right)\log\left(1-{ŷ}_{2D-3D}^{\left(i\right)}\right) \end{array}$$

where *n* is the number of samples and *y*^(*i*)^ is the ground-truth label of the *i*th sample. ${ŷ}_{2D-SADN}^{\left(i\right)}$, ${ŷ}_{3D-DGDN}^{\left(i\right)}$, and ${ŷ}_{2D-3D}^{\left(i\right)}$ are the *i*th output value of sigmoid activation of 2D-SADN, 3D-DGDN, and the integrated network, respectively. ${ŷ}_{2D-3D}^{\left(i\right)}$ is computed by logit values of network 2D-SADN and 3D-DGDN as follows:

(11) $$\begin{array}{r} {ŷ}_{2D-3D}^{\left(i\right)}=\sigma \left(l_{2D-SADN}^{\left(i\right)}+l_{3D-DGDN}^{\left(i\right)}\right) \end{array}$$

where $l_{2D-SADN}^{\left(i\right)}$ and $l_{3D-DGDN}^{\left(i\right)}$ are the *i*th logit values of network 2D-SADN and 3D-DGDN, respectively. σ(∙) is a sigmoid activation function.

(12) $$\begin{array}{r} l_{k}^{\left(i\right)}=\log{\left(\frac{x_{i}}{1-x_{i}}\right)}_{k}\;\forall x_{i}\in \left(0,1\right) \end{array}$$

where *k* means network 2D-SADN and 3D-DGDN, and *x*_*i*_ is the *i*th output value of sigmoid of network *k*. The final prediction is the index with the maximum value from the output of sigmoid of the integrated network as follows:

(13) $$\begin{array}{r} \hat{P}=\underset{i}{\mathrm{argmax}}{ŷ}_{2D-3D}^{\left(i\right)} \end{array}$$

The paper uses 10-fold cross-validation to evaluate experiments for excluding the differences caused by individuality and over-fitting. Note that 80% of samples from the total participants are used for training, 10% for validation, and the rest of 10% for testing. Each fold includes the data from 42 participants for training, 5 participants for validation, and 5 participants for testing. We use accuracy to evaluate the proposed model performance. Accuracy is computed by the confusion matrix consisting of the number of true positives (*TP*), true negatives (*TN*), false positives (*FP*), and false negative (*FN*), defined as follows:

(14) $$\begin{array}{r} \mathrm{accuracy}=\frac{TP+TN}{TP+TN+FP+FN} \end{array}$$

where *TP* is the number of depression samples predicted to be depressed, *TN* is the number of healthy samples predicted to be healthy, *FP* is the number of healthy samples predicted to be depression, and *FN* is the number of depressed samples predicted to be healthy.

## 4. Experiments

### 4.1. Depression Data Collection

To effectively obtain depression data, we cooperated with Tianshui Third People's Hospital in Gansu province to collect data. Data collection was accomplished in an isolated, quiet, and soundproof room without electromagnetic interference. Two people were present in the room at the same time: one was the clinician controlling the data collection process, and the other was the participant. The clinician operated one of the two computers and played all the stimulus tasks (film clips, voice responses, text reading, and picture description) sequentially. The stimulus materials were displayed to the participant on the second computer. Participants must evaluate their emotional state before and after completing each stimulus task. Each stimulus task was divided into positive, neutral, and negative stimuli. In order to prevent the stimulus of the previous emotional valence from affecting the next emotional valence stimulus, there is a 1-min break at the end of each material. Moreover, the order of valence stimulation presented to each subject was also different. Audio and video information on the participant was recorded by a webcam, a Kinect camera, and a microphone.

#### 4.1.1. Participants

Every participant is rated by psychiatrists through interviews and questionnaires. The set of questionnaires required to be filled included the International Neuropsychiatric Interview (MINI) and the Patient Health Questionnaire-9 (PHQ-9). PHQ-9 was the main grouping criteria (health control: <5, patient: ≥5). PHQ-9 scores are treated as the label. In this experiment, the out-patient sample set included data from 52 males and 52 females; meanwhile, the control group also included data from 52 males and 52 females. Participants were excluded from the health group if they received a Beck depression inventory (BDI) score >5. The demographic characteristics of all participants are shown in Table 1. All participants provided informed consent.

**Table 1:** Demographic characteristic of the out-patients and control group: mean and (standard deviation).

**Gender**	**Category**	**Number**	**Age**	**Education**	**PHQ-9**	**BDI**
Male	Control group	52	39 (10.8)	11.8 (2.5)	0.8 (2.0)	6.4 (6.4)
	Out-patients	52	34.8 (11.1)	11.2 (3.4)	17.5 (5.6)	26.4 (12.8)
Female	Control group	52	34.7 (10.7)	12.3 (3.2)	0.3 (0.7)	4.7 (5.3)
	Out-patients	52	37.4 (10.4)	10.8 (4.0)	18.3 (5.6)	33.5 (13.2)

#### 4.1.2. Paradigm Design of Depression Experiments

Depressed individuals have negative self-schema in cognitive processing related to attention control disorder of emotional interference. The phenomena related to emotion include subjective experience, facial expression behavior, individual differences in nervous system response, and emotional response. In order to obtain useful data, we need to choose appropriate emotional induction methods. Using the classic oddball experimental paradigm of psychology (Li, 2014), we designed 5 stimuli tasks of 3 emotional valences to induce behavioral differences between healthy and depressed individuals. The tasks included:

1. *Watching film clips:* Three short film segments around 90 s each, one positive, another neutral, and the other negative, were disciplinarily presented. Participants were asked to watch the film clip and then describe their mood. The clips had previously been rated for their affective content (Gross and Levenson, 1995). The positive film clip is excerpted from cartoon “Larva Funny Bugs,” the neutral film clip is excerpted from the documentary “Universe Millennium,” and the negative film clip is excerpted from the movie “October Siege.” The synchronized start of the stimuli with the recording enabled us to draw a correlation between facial activity and the stimuli.
2. *Replying to nine free-response questions:* Each participant was requested to respond to nine specific questions (three positives, three neutrals, and three negatives). These questions are designed based on DSM-IV and other depression scales such as the Hamilton depression rating scale (HDRS)^2^. Questions included, for instance, “what kind of lifestyle do you like?” “discuss a sad childhood memory,” and “please evaluate yourself.” The answers were synchronized with the facial activity recorded.
3. *Reading three phonetically balanced passages containing affective content:* The participants were presented with a paragraph of text on a computer screen and asked to finish the reading as naturally as possible. There are three reading materials. One of passages contained positive words (e.g., glorious, victory), and the other contained negative words (e.g., heart-broken, pain), which were selected from affective the ontology corpus created by Hongfei Lin^3^. The last one included neutral words (e.g., village, center) selected from the extremum table of affective Chinese words (Gong et al., 2011). The reading and recording commenced synchronously.
4. *Describing pictures:* The picture description section is to present 6 pictures in sequence on the computer screen. The first three pictures are facial expression pictures of three women divided into positive, neutral, and negative, and the last three pictures are three scene pictures divided into positive, neutral, and negative. All pictures were selected from the Chinese Facial Affective Picture System (CFAPS) (Gong et al., 2011). Participants were requested to observe the picture and then describe it. Reporting logs enabled correlations between image presentations and facial activity to be established.

#### 4.1.3. Process of Affective Rating

**Figure 6:**
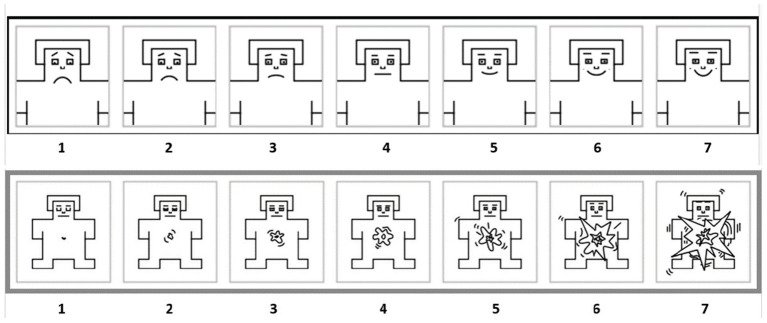
Schematic diagram of affective rating. The top is valence rating. The bottom is arousal rating.

The Self-Assessment Manikin (SAM) (Lang, 1980) which is an affective rating-scale system using a graphical figure that depicts the dimensions of valence (from a smiling figure to a frowning figure) and arousal (from an excited to a relaxed figure), is used to measure a participant's emotion, as shown in Figure 6. The affective rating process consists of three parts: emotional pretest, emotion-eliciting tasks, and emotional posttest.

#### 4.1.4. Constructing Depression Dataset

**Figure 7:**
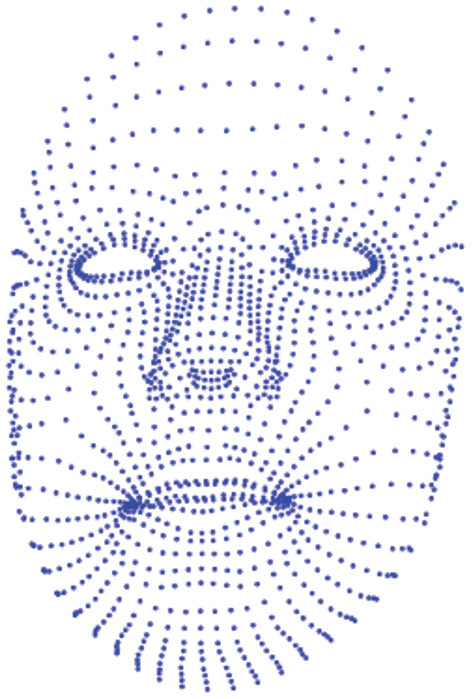
Facial contour that consists of 1,347 vertices generated by Kinect.

Every participant has to complete five stimulus tasks of three emotional valences in turn, resulting in 15 datasets: three datasets for watching film clips (one positive + one neutral + one negative), three datasets for nine interviews (three positive + three neutral + three negative), three datasets for text readings (one positive + one neutral + one negative), three datasets for expression image descriptions (one positive + one neutral + one negative), and three datasets for scene image descriptions (one positive + one neutral + one negative). The database consists of four folders for each participant, which are voice, video, emotional state, and information log. Fifteen monophonic speech recordings are made in the voice folder. A sampling rate of 44.1 kHz and a sampling depth of 24-bit are used for collecting speech signals. Speech recordings are saved in the uncompressed WAV format. Ambient noise should be lower than 60 dB. There are two types of data in the video folder. One is 15 video recordings of 640 × 480 pixel, 30 fps collected by a webcam, and saved as mp4; the other is 15 recordings obtained by the Kinect, and each recording contains two kinds of data: three-dimensional coordinates of 1,347 facial points and 17 AUs. Every facial point is a 3D point with X, Y, and Z coordinates. Figure 7 shows that facial contours consist of 1,347 feature points. At present, many AU detection methods use feature point tracking, shape modeling, template matching, and neural network to recognize the AU features of the face. In this paper, the Kinect device automatically recognizes the AU of the face through the built-in API interface and the facial AU detection algorithm. The intensity of each AU in each frame was calculated. The intensity amplitude was between -1 and 1, and the facial expression was directly measured by digital features. Seventeen AUs recorded by Kinect are corresponding to AUs encoded by FACS. AU from Kinect can appear separately or in combination to show different expressions^4^. Fifteen three-dimensional facial points recordings and 15 AUs recordings are saved as CSV format. Participants are assessed for the two dimensions of valence and arousal before and after receiving different emotion-eliciting tasks to obtain 15 evaluation results, which are saved in the emotional state folder. The information related to the subjects is saved in the information log, which are name, gender, age, profession, education background, label, PHQ-9, BDI, and so on.

### 4.2. Data Pre-processing

We intercepted the data which the subjects did not speak during the three tasks of watching the film clips, facial expression pictures description, and scene pictures description. The machine learning toolkit Dlib (King, 2009) is used to acquire facial region cropped and aligned according to each video's eye location. All images are resized to 100 × 100. Considering the problem of many frame redundancy in the video clip, we adopt the sampling scheme of taking one frame every 100 frames. Experiments determine the sampling interval according to the number of frames or length of each video and the frequency of facial changes. Finally, we obtained 156,000 facial image data in any given stimulus task. Then, we adopt the same sampling strategy on the Kinect dataset to ensure that the two data are aligned on the time axis. These data were used for training, validation, and testing networks. The original image is then divided into three images by R, G, and B channels and used to train a DBN. Every image is flatted, and then the pixel value of which is normalized for input in 2D-SADN. In 3D-DGDN, three-dimensional coordinates of 1,347 facial points can be considered as one-dimension vector at frame t and defined as ${\left[x_{1}^{\left(t\right)}y_{1}^{\left(t\right)}z_{1}^{\left(t\right)}\cdots x_{n}^{\left(t\right)}y_{n}^{\left(t\right)}z_{n}^{\left(t\right)}\right]}^{T}$, where *n* is the total number of landmark points at frame *t*. *I*−*score* standardization is used to normalize xyz-coordinates as follows:

(15) $$\begin{array}{r} {\bar{x}}_{i}^{\left(t\right)}=\frac{x_{i}^{\left(t\right)}-\mu }{\sigma } \end{array}$$

where $x_{i}^{\left(t\right)}$ is x-coordinate of the *i*th facial landmark point at frame *t*, μ, and σ is mean value and standard deviation of x-coordinate at frame *t*, respectively. This process is also applied to $y_{i}^{\left(t\right)}$, $z_{i}^{\left(t\right)}$, and AUs. Finally, these normalized points are concatenated ${\left[{\bar{x}}_{1}^{\left(t\right)}{ȳ}_{1}^{\left(t\right)}{\bar{z}}_{1}^{\left(t\right)}\cdots {\bar{x}}_{n}^{\left(t\right)}{ȳ}_{n}^{\left(t\right)}{\bar{z}}_{n}^{\left(t\right)}\right]}^{T}$ or ${\left[{\bar{x}}_{1}^{\left(t\right)}{ȳ}_{1}^{\left(t\right)}{\bar{z}}_{1}^{\left(t\right)}\cdots {\bar{x}}_{n}^{\left(t\right)}{ȳ}_{n}^{\left(t\right)}{\bar{z}}_{n}^{\left(t\right)}AU_{1}^{\left(t\right)}\cdots AU_{17}^{\left(t\right)}\right]}^{T}$ to input the DBN.

### 4.3. Network Architecture

The DBNs structure of 2D-SADN and 3D-DGDN are similar. The first layer RBM is trained completely unsupervised for all DBNs. The biases and weights are randomly sampled from a normal distribution with μ = 0, σ = 0.01. They are all updated after a full minibatch. Because the preprocessed data is larger in 2D-SADN, a penalty term is added to the weight and bias updates to obtain sparse representation. λ is fixed as 3, the sparsity parameter of bias is 0.1, and the learning rate is 0.02. The rest RBMs are also trained for 100 epochs using the same value used for training the first level RBM for all DBNs.

The hidden nodes number of every channel DBN is fixed to 8192-4096-2048-502 in 2D-SADN. The number of hidden nodes for 4DBN and 4DBN-AU from the first layer to 4-layer is selected over 3000-2048-1024-128 in 3D-DGDN. However, for AU-4DBN, the penultimate hidden layer with 1,024 nodes similar to 4DBN is then concatenated with AUs, and the resulting input serves as the visible layer of a top-level RBM with 150 hidden nodes (Gaussian–Bernoulli).

Our LSTM has two layers, one with 200 nodes and another with 64 nodes. We initialize the hidden states to zero and then use the current minibatch's final hidden states as the initial hidden state of the subsequent minibatch. The batch size is 50, and the training epoch is 50. The learning rate is set by grid search, and the momentum is 0.9.

The whole system is tested on the TensorFlow deep learning framework with a Xeon(R) CPU E7-4820 v4@2.00 GHz processor, 128 Gigabytes memory, and a Telsa M60 GPU, which can meet our computing needs.

### 4.4. Experimental Results

#### 4.4.1. Qualitative Analysis of Stimulus Tasks

Previous studies have shown that depressed patients have less positive emotions and more negative emotions than healthy individuals, which indicates that depressed patients show sad when stimulated by positive or negative emotions (Delle-Vigne et al., 2014). Depressed patients will be less sensitive to emotional stimuli from the outside world; that is to say, it is difficult for depressed patients to elicit corresponding emotional feedback (Rottenberg, 2005). The differences in valence and arousal dimensions between the healthy and depressed groups for the five stimuli with three emotional valences were calculated, respectively, as shown in Table 2.

**Table 2:** The differences in valence and arouse dimension between the healthy and depressed group for the five stimuli with three emotional valences (*P* < 0.1).

**Emotional dimension**	**Tasks**	**Subjects**	**Positive**	**Neutral**	**Negative**
	Film	Health	1.635	0.074	–1.534
		depression	–1.058	–0.036	–1.078
		*P*-value	**0.043**	2.428	**0.052**
	Question	Health	1.356	0.088	–0.744
		Depression	–0.273	0.333	–0.330
		*P*-value	0.601	0.565	1.035
Valence	Reading	Health	0.947	0.260	0.829
		Depression	0.273	0.242	0.333
		*P*-value	0.233	0.137	0.531
	Expression figure	Health	1.084	0.205	–0.938
		Depression	–0.152	–0.198	–0.506
		*P*-value	**0.073**	0.960	**0.085**
	Scene figure	Health	0.874	0.110	–0.123
		Depression	–0.015	0.061	0.303
		*P*-value	0.125	1.531	0.211
	Film	Health	1.058	–0.205	1.045
		Depression	–0.635	0.076	0.014
		*P*-value	**0.072**	0.151	**0.065**
	Question	Health	0.968	0.027	0.109
		Depression	–0.060	–0.030	0.151
		*P*-value	0.254	**0.096**	0.325
arousal	Reading	Health	–0.810	0.137	0.164
		Depression	–0.182	–0.106	0.076
		*P*-value	0.222	0.115	0.177
	Expression figure	Health	1.008	0.205	0.233
		Depression	–0.014	0.333	0.106
		*P*-value	**0.098**	**0.042**	**0.087**
	Scene figure	Health	0.219	0.055	–0.068
		Depression	0.015	–0.091	–0.121
		*P*-value	0.146	**0.033**	**0.088**

*The table's data are the statistical value of the difference between the pretest and posttest of the valence dimension and arouse dimension under different stimuli tasks*.

*Bold indicates significant difference*.

From Table 2, we can find that the absolute value of the healthy group's valence difference is generally greater than that of the depressed group in all stimulus tasks. The valence difference of the healthy group is basically consistent with emotional valence, which means that positive tasks stimulate joyful emotions, negative tasks stimulate sad emotions for the healthy group (the valence difference in positive stimuli is positive, and the valence difference in negative stimuli is negative), but for the depressed group, both positive and negative stimuli basically arouse sad emotions (the valence difference in positive and negative stimuli is negative). From the *T*-test values, it can be found that there is a significant difference in valence between the healthy group and depressed group under positive and negative stimulation, especially in the stimulation tasks of film clips and characters' facial expressions, as shown in bold.

From Table 2, we also can find that the arousal difference of the healthy group is almost greater than that of the depressed group in all stimulus tasks, and the arousal difference of the healthy group is basically positive, which means that the healthy group is more likely to be aroused than the depressed group. From the *T*-test values, it can be found that there is a significant difference in arousal between the healthy group and depressed group under positive and negative stimulation, especially in the stimulation tasks of film clips and characters' facial expressions, as shown in bold.

**Figure 8:**
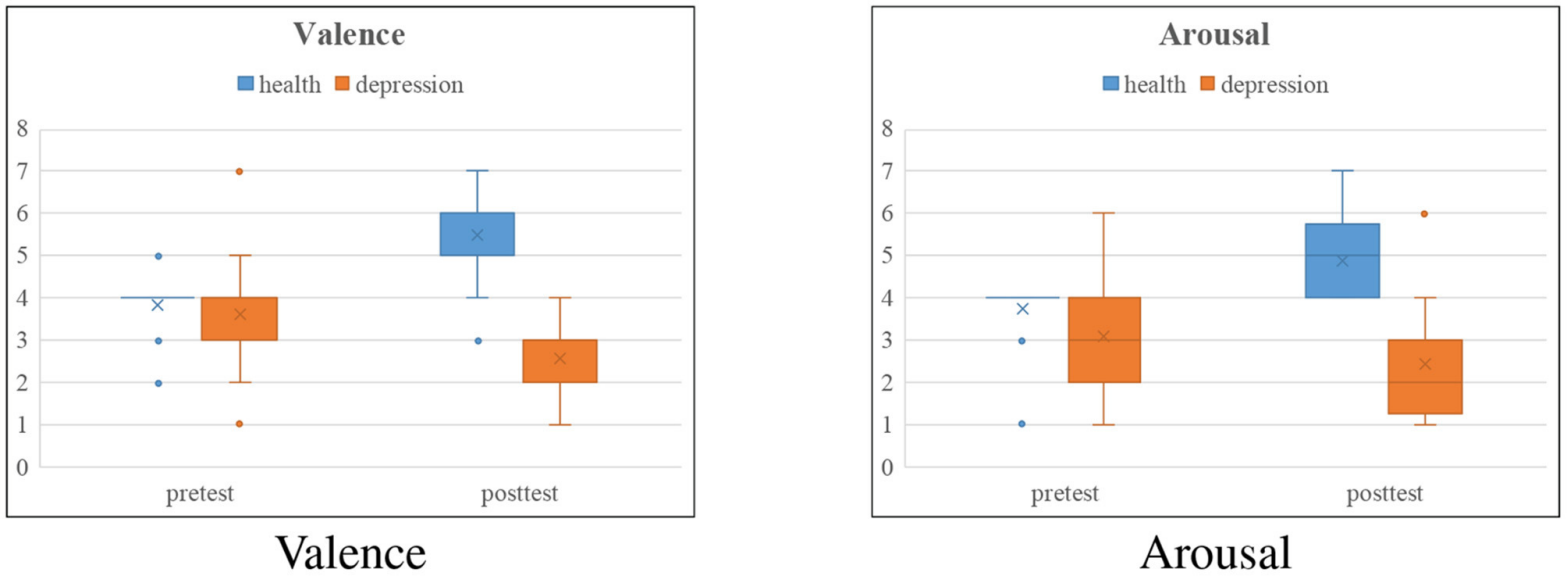
Box comparison charts of the valence and arousal of the healthy group and depressed group before and after positive film clips stimulation.

From Table 2, we can draw the following conclusions: positive and negative film clips and facial expression pictures are more likely to inspire significant differences between the healthy and depressed groups than the other three tasks. Moreover, the results reflected from Table 2 are also consistent with paper (Delle-Vigne et al., 2014) and (Rottenberg, 2005). In order to more intuitively reflect the effectiveness of the experimental paradigm we designed, we draw comparison charts of the valence and arousal of the healthy group and depressed group before and after positive film clips stimulation, as shown in Figure 8. The healthy group was in a calm mood before watching the positive film clip, and the pleasure degree increased significantly after watching the film clip, which stimulated a happy mood. The depressed group felt a little sad before watching the film clip, but their mood became more and more depressed after watching the film clip, and the arousal degree did not change much. This is consistent with the characteristics of depression.

#### 4.4.2. Determining the Number of Network Layers

**Figure 9:**
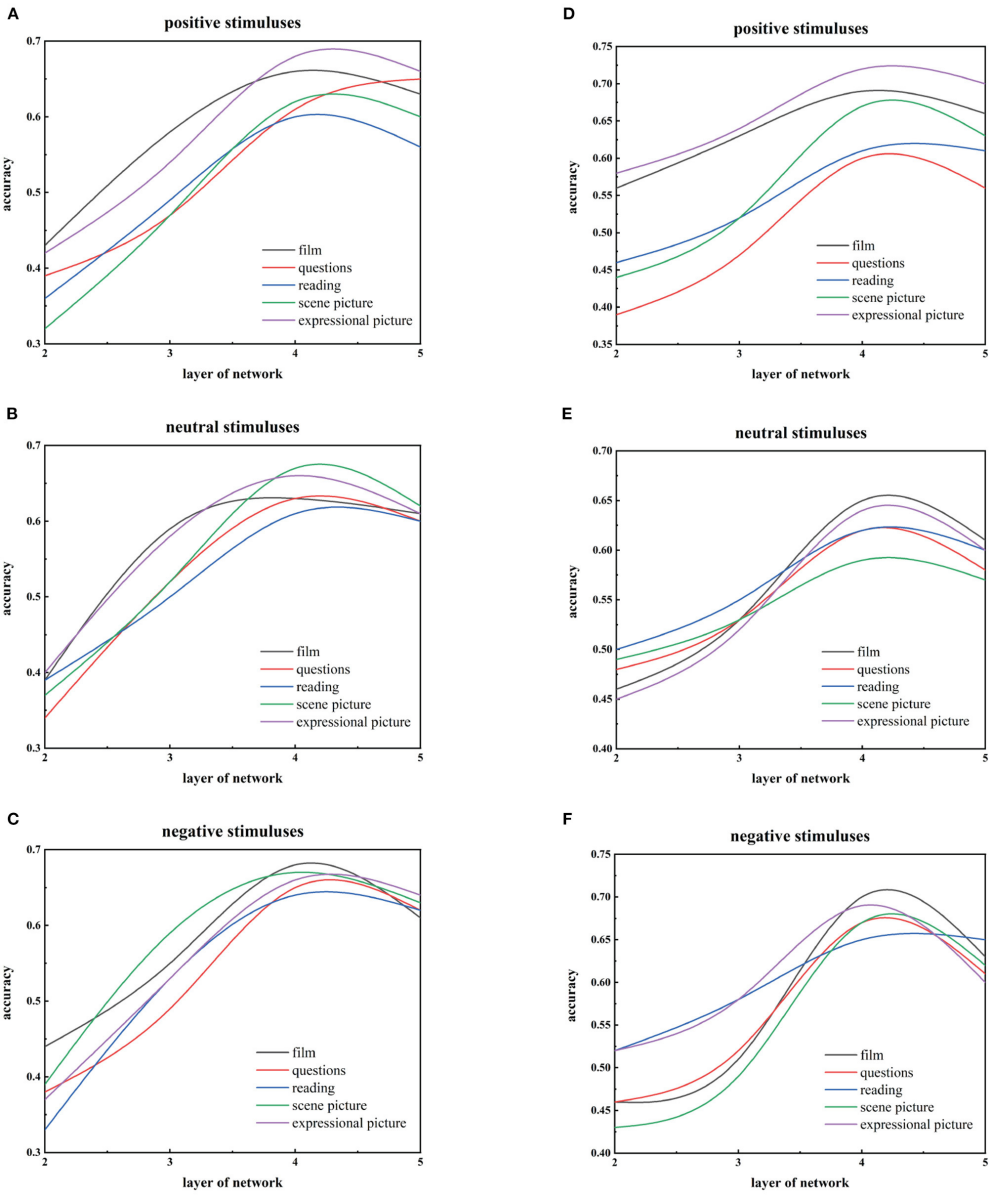
Comparison of recognition performance with the different hidden levels on three emotional valences of the five stimuli for 3D facial points and 2D images. **(A–C)** are based on 3D facial points. **(D–F)** are based on 2D images.

We use three kinds of data in the whole framework, namely 2D images, 3D facial landmark points, and AUs. We first use 2D face images and 3D facial landmark points as input to train different deep DBNs for the five stimuli with three emotional valences, respectively. We use the validation set to test networks and find that the recognition accuracy of the both mainly increases with the number of layers, reaching the highest on the forth hidden layer using 2D images or 3D landmark points trained DBN models, but both of them subsequently lose recognition performance as the number of layers increases, as shown in Figure 9. Therefore, we regard 4-hidden-layer DBN as a benchmark model of the 3D-DGDN.

#### 4.4.3. Global Performance Analysis

**Figure 10:**
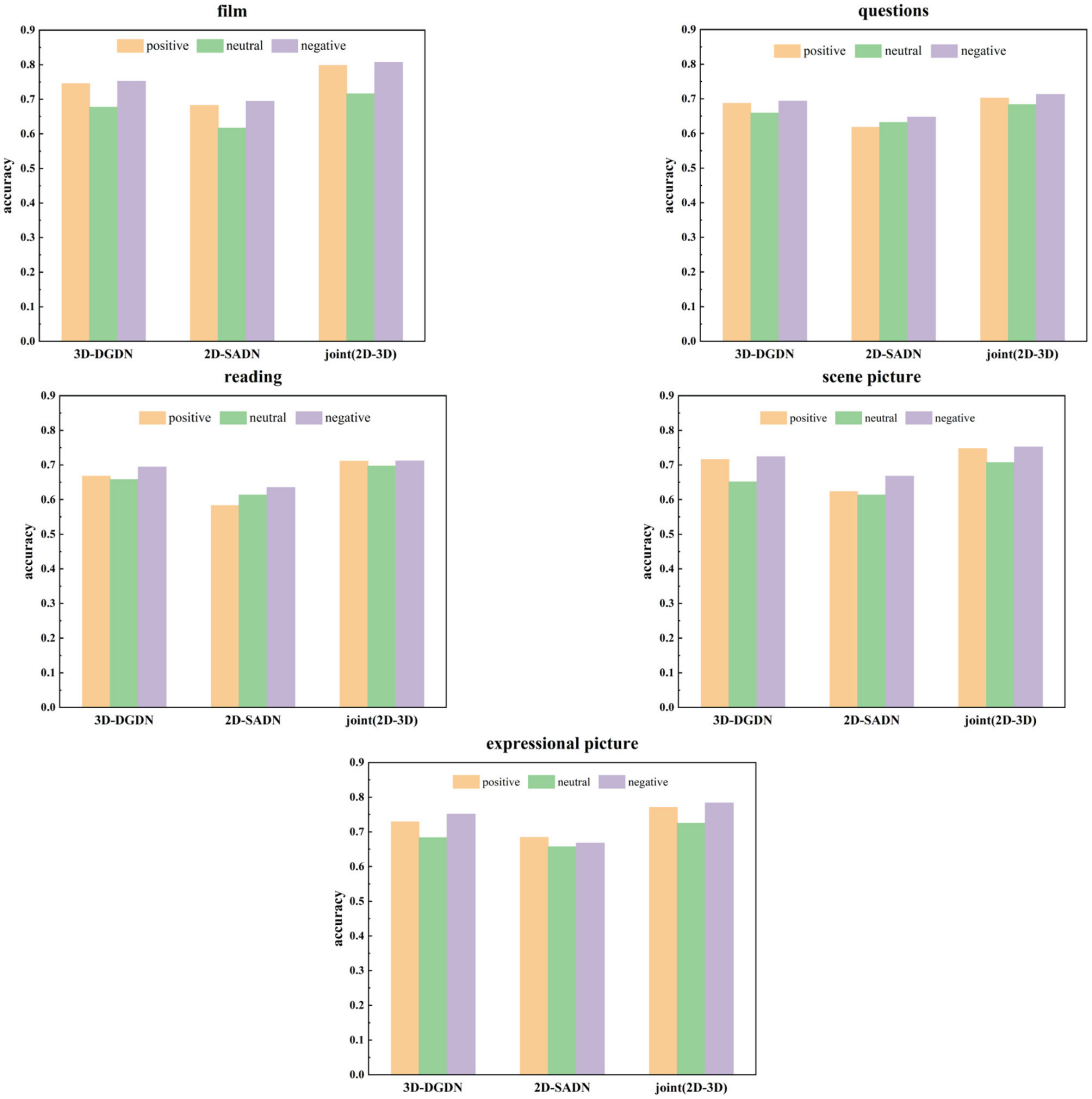
Comparison of accuracy with the five stimuli among three networks.

The accuracy of all proposed models for three emotional valences of the five stimuli was tested on the dataset, including all males and females. The experiment results are shown in Table 3 and Figure 10. It can be seen from Table 3 and Figure 10 that the best performance among the three emotional valences of the five stimuli was obtained based on the joint model. In particular, the performance of 3D-DGDN was higher than the 2D-SADN in all tasks. In the process of data collection, it was found that depressed patients or subjects with depressive tendencies were more prone to hyperactivity, which would lead to changes in depth information. Therefore, we added time series information to depth information for modeling, which will obtain more discriminative features. However, the combined network produced good results. This indicates that the 2D-SADN network is a performance supplement to the 3D-DGDN network, and the two networks are complementary to each other.

**Table 3:** The accuracy of all models for three emotional valences of five stimuli.

**Type**	**Models**	**Positive**	**Neutral**	**Negative**	**Mean**
	4DBN-AU	0.638	0.603	0.673	0.638
	AU-4DBN	0.693	0.635	0.701	0.676
Film	3D-DGDN	0.745	0.677	0.752	0.725
	2D-SADN	0.682	0.617	0.694	0.664
	Joint(2D-3D)	**0.798**	0.716	**0.807**	**0.774**
	4DBN-AU	0.605	0.593	0.592	0.597
	AU-4DBN	0.639	0.636	0.642	0.639
Question	3D-DGDN	0.687	0.659	0.693	0.680
	2D-SADN	0.618	0.632	0.647	0.632
	Joint(2D-3D)	0.702	0.683	0.713	0.699
	4DBN-AU	0.572	0.583	0.601	0.585
	AU-4DBN	0.623	0.625	0.652	0.633
Reading	3D-DGDN	0.668	0.658	0.694	0.673
	2D-SADN	0.583	0.613	0.635	0.610
	Joint(2D-3D)	0.711	0.697	0.712	0.707
	4DBN-AU	0.617	0.538	0.608	0.588
	AU-4DBN	0.671	0.592	0.672	0.645
Scene picture	3D-DGDN	0.716	0.651	0.724	0.697
	2D-SADN	0.623	0.613	0.668	0.635
	Joint(2D-3D)	0.747	0.707	0.752	0.735
	4DBN-AU	0.659	0.591	0.635	0.628
	AU-4DBN	0.703	0.642	0.690	0.678
Expression picture	3D-DGDN	0.729	0.683	0.751	0.721
	2D-SADN	0.684	0.657	0.668	0.700
	Joint(2D-3D)	**0.770**	0.725	**0.783**	**0.759**

*Bold indicates a higher recognition rate*.

It can be also seen that the recognition accuracy of positive and negative stimuli is higher than that of neutral stimulus, which is consistent with the emotional feedback theory of depressed patients compared with the healthy group, patients with depression have behavioral patterns such as weakened positive emotional feedback and enhanced negative emotional feedback. So in some cases, they have formed specific facial expressions such as reduced positive expressions and increased negative expressions (Babette et al., 2005). That is to say, they will not produce a larger change in expression compared with the normal population when facing the same stimulus. Therefore, there is a significant difference between positive and negative stimuli. Simultaneously, the accuracy of watching film clips is relatively higher in all positive or negative stimulus tasks and the highest recognition rates reach up to 0.798 and 0.807 for positive and negative stimulus, respectively, as shown in bold. The next high recognition rate is to view the expressional picture, as shown in bold. Because emotionally charged clips and images can, in principle, elicit an observable response (Pampouchidou et al., 2019). It is because that in order to eliminate the influence of unrelated facial movements on the facial expression behavior analysis of the participants, we only used the experimental data that the participants are completely prohibited from speaking in these two tasks. Relatively poor accuracies are obtained for questions and readings, which could be because facial expressions are associated with speech. When one feature is mixed with other factors, the purity expressed by this feature is not high.

#### 4.4.4. Difference in Gender Analysis

**Figure 11:**
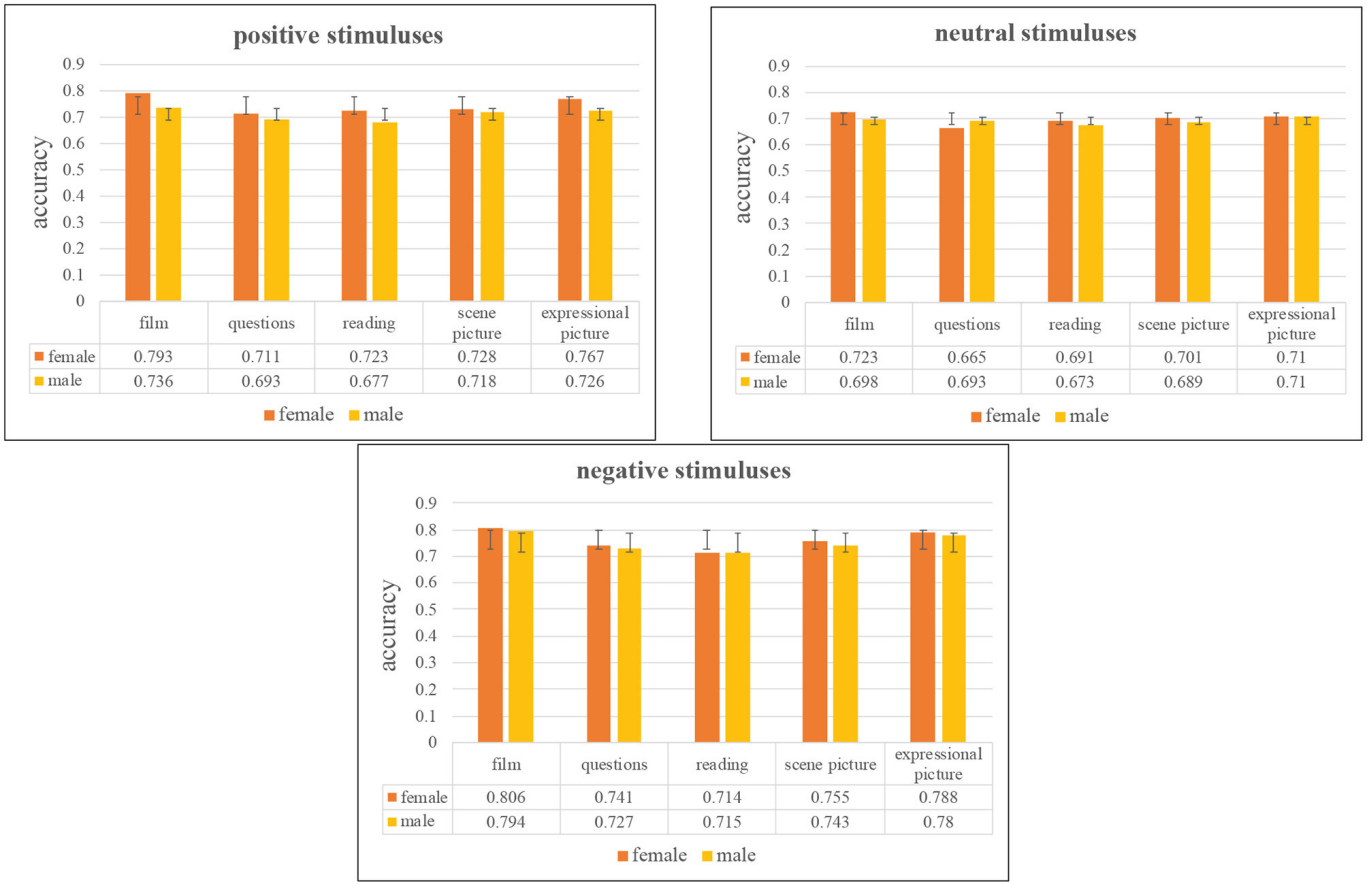
Comparison of accuracy on different gender under 95% confidence interval.

The gender-dependent experiments are analyzed based on the integrated network. Figure 11 shows the comparison of depression recognition based on gender difference under 95% confidence interval. From Figure 11, we can find that females' recognition accuracies are universally higher than that of males in three kinds of emotion valence, which explains that females are more likely to be aroused emotionally. According to WHO, evidence suggests that women are more prone than men to experience anxiety, depression, and somatic complaints—physical symptoms that cannot be explained medically^5^. We can also see that women are more likely to show the effects of positive stimulation than men. Among the three emotional valence tasks, the difference between female and male groups under the negative stimuluses are the smallest, which indicates that both female and male groups have higher accuracy and sensitivity under the negative stimuluses. In general, female are more emotionally aroused than male.

#### 4.4.5. Comparative Analysis of Relevant Works

We compared our method's accuracy with the methods using the same data set, as shown in Table 4. Although our accuracy rate was slightly lower than that in previous studies (Li et al., 2018) (the selected samples are all major depressive disorder) in neutral stimulation tasks, we have improved the accuracy in the remaining tasks. Studies have shown that severely depressed individuals have lower emotional feedback to both positive and negative stimuli compared with healthy individuals (Nesse and Randolph, 2000). Therefore, our research results are more in line with the emotional experience of depressed patients. However, the performance has declined compared with our previous work (Guo et al., 2019). We will compare and summarize from the following points:

- *Data source:* The previous work only used the three-dimensional face point data collected by Kinect, although the point data can better handle some additional variables in the picture, such as illumination, angle, skin color, and so on, the videos (pictures) collected by the optical camera are still the main focus in actual application scenarios. Therefore, we combined the two data sources to make up for each other in this work.
- *Model:* The main framework of the DBN model was used in the two works. Considering that it was an extension of the previous work, the same data samples and the main model framework were used. However, this work further upgraded the model, using DBN to extract static facia appearance information and combined with the LSTM network to extract the dynamic information, and finally through a full connection to connect the two networks. The design of the model is complete and more prosperous than the previous work.
- *Practice:* We sampled frames by frames in the previous work, preprocessed 3D facial points data, and converted them into 200*200 grayscale images. It took nearly a week to calculate the entire batch of data for 30 cycles. In this work, we sampled between frames and processed the original data directly. Although the network was more complicated than previous work, the calculation time was reduced. It only took 5 days to complete the whole batch of data for 100 cycles.

**Table 4:** Comparison of accuracy based on the same database.

**Gender**	**Task**	**Accuracy**								
		**Positive stimulus**			**Neutral stimulus**			**Negative stimulus**		
		**This work**	**Previous work**	**(Li et al., 2018)**	**This work**	**Previous work**	**(Li et al., 2018)**	**This work**	**Previous work**	**(Li et al., 2018)**
	Film clips	0.793	0.825	0.737	0.723	0.761	0.868	0.806	0.816	0.763
	Questions	0.711	0.761	0.649	0.665	0.705	0.737	0.741	0.765	0.675
Female	Readings	0.723	0.768	0.711	0.691	0.728	0.658	0.714	0.751	0.658
	Scene pictures	0.728	0.801	0.711	0.701	0.713	0.763	0.755	0.806	0.711
	Expression pictures	0.767	0.806	0.632	0.710	0.741	0.737	0.788	0.801	0.711
	Film clips	0.736	0.772	0.647	0.698	0.733	0.794	0.794	0.782	0.647
	Questions	0.693	0.738	0.725	0.693	0.728	0.735	0.727	0.726	0.667
Male	Readings	0.677	0.724	0.618	0.673	0.694	0.676	0.715	0.745	0.588
	Scene pictures	0.718	0.755	0.618	0.689	0.714	0.706	0.743	0.776	0.647
	Expression pictures	0.726	0.761	0.647	0.710	0.673	0.706	0.780	0.737	0.588

However, the classification accuracy has declined due to the following aspects.

First of all, the previous work results showed that visual stimuli classification effect, such as watching film clips and pictures, was better than the classification effect of language expression (here, mainly talking about changes in facial expressions). In our entire experiment, subjects completed every task and were asked to answer questions. The data used earlier included responses to questions on watching the film clips and seeing facial/scene expression tasks. In this work, to further verify whether direct visual stimuli are more likely to elicit emotions in the depressed group, we selected only the data participants did not speak during watching the film clips and seeing facial/scene expression tasks. Coupled with the strategy of sampling between frames, the overall amount of data is far less than the previous work. For deep learning, the more considerable the amount of data, the better the trained model's performance.

Then, we converted 1,347 three-dimensional points into 200*200 grayscale images in the preliminary work, which undoubtedly added some additional information for recognition. Whether this information played a role in the performance of the model could not be reasonably explained. In this work, we directly used the raw data without any additional information, so the final results are calculated from both the data and the model.

Besides, this paper's conclusion was obtained after 100 cycles based on 10-fold cross-validation, which is more stable than previous work.

In conclusion, on the basis of using the original data, the study in this paper significantly reduces the calculation time, while the accuracy rate is slightly lower than before, such as 0.02 for women and 0.01 for men. Based on the above points, this work is more reasonable and universal from three aspects of theory, model, and practice.

## 5. Conclusion and Future Work

Depression is a common mental illness that can negatively affect people's mental health and daily life. In recent years, researchers have been looking for an objective evaluation method and quantitative indicators to identify depression objectively and effectively. Among them, the research of depression recognition based on facial expression behavior is a hot topic. In this paper, We first designed an experimental paradigm that can effectively stimulate the emotional differences between healthy and depressed groups and established a database for identifying depression. And then, we presented two deep network models that collaborate with each other. The first network was 2D-SADN, which is used to extract the static appearance features from images, and the second network was 3D-DGDN, which captures the dynamic geometry features of 3D facial landmark points and AUs from Kinect. We showed that the accuracy obtained by the 2D-SADN was lower than that of the 3D-DGDN, which may be because poor image quality and the DBN model cannot well retain the 2D information of an image. At last, we achieved the best recognition rates using the integrated deep network on the collected databases.

From the perspective of emotional stimulus materials, the experimental results also support this theory: apparent differences existed between the health and depressed groups for pleasant or unpleasant stimuli. Mostly, the accuracy of watching film clips and expressional pictures emotional stimulus tasks were generally high, but the accuracy of answering questions and reading texts is low. This is because the subjects recorded facial expressions while speaking in both the masks, with one feature being mixed with other factors. We will further investigate the experimental strategies to construct a more distinctively characteristic depression behavior database in future work. We will further analyze which of the two states of speech and non-speech information on discriminating depressed patients. Since CNN has shown superior performance on image classification/recognition problem, we aim to use the CNN-based methods to model depth information and video information. We also will try to use the state-of-the-art multimodal fusion methods to identify depression.

## Data Availability Statement

The data analyzed in this study is subject to the following licenses/restrictions: Data involves privacy and has not been disclosed. Requests to access these datasets should be directed to Zhenyu Liu, liuzhenyu@lzu.edu.cn.

## Ethics Statement

The studies involving human participants were reviewed and approved by Tianshui Third People's Hospital and Lanzhou University. The patients/participants provided their written informed consent to participate in this study.

## Author Contributions

All authors listed have made a substantial, direct, and intellectual contribution to the work. WG, HY, and BH were responsible for the entire study, including study concepts and study design. WG, ZL, and YX contributed to the experimental paradigm design. WG and YX were responsible for collecting data. WG wrote the manuscript. HY helped WG draft the manuscript and modify the important content. All authors read and approved the final manuscript.

## Conflict of Interest

The authors declare that the research was conducted in the absence of any commercial or financial relationships that could be construed as a potential conflict of interest.

## Footnotes

^1^Available at: http://medlineplus.gov/depress.html.

^2^Available at: http://ir.dlut.edu.cn/Group.aspx?ID=4.

^3^Available at: http://www.datatang.com/data/43216.

^4^Available at: https://blog.csdn.net/u014365862/article/details/48212757.

^5^Available at: https://www.who.int/life-course/news/commentaries/2015-intl-womens-day/.
