# Supplementary material for: Deep Neural Networks for Depression Recognition Based on 2D and 3D Facial Expressions Under Emotional Stimulus Tasks
Source: Front Neurosci. 2021 Apr 23;15:609760. doi: 10.3389/fnins.2021.609760 (PMC8102822; doi:10.3389/fnins.2021.609760)
Supplement: Supplementary file 1 [file Table_1.pdf]

Supplementary Material

SUPPLEMENTARY TABLE

17 AUs recorded by Kinect are corresponding to AUs encoded by FACS. The specific meaning is shown in Table:

Table AU Definition and intensity range collected by Kinect device

Kinect AU No	Description	intensity range	FACS AU No
1	Jaw open	[0,1]	26
2	Lip pucker	[0,1]	18
3	Jaw slide right	[-1,1]	30
4	Lip stretcher right	[0,1]	20
5	Lip stretcher left	[0,1]	20
6	Lip corner puller left	[0,1]	12
7	Lip corner puller right	[0,1]	12
8	Lip corner depressor left	[0,1]	15
9	Lip corner depressor right	[0,1]	15
10	Left cheek puff	[0,1]	34
11	Right cheek puff	[0,1]	34
12	Left eye closed	[0,1]	43
13	right eye closed	[0,1]	43
14	right eyebrow lowerer	[-1,1]	4
15	left eyebrow lowerer	[-1,1]	4
16	lower lip depressor left	[0,1]	16
17	lower lip depressor right	[0,1]	16

It can be seen that AU from Kinect can appear separately or in combination. When action units are combined, different facial expressions will be involved. For example, AU1, AU4, AU5, AU6, AU7 combination will show happy expressions, and AU8, AU9, AU14, AU15, AU16, AU17 combination will show sad expressions. Therefore, AU's amplitude intensity is correlated with the expression behavior, which is also a good quantitative indicator.
